# miRNA-145 inhibits non-small cell lung cancer cell proliferation by targeting c-Myc

**DOI:** 10.1186/1756-9966-29-151

**Published:** 2010-11-22

**Authors:** Zhe Chen, Huazong Zeng, Yong Guo, Pei Liu, Hui Pan, Anmei Deng, Jian Hu

**Affiliations:** 1 National Clinical Research Base of Traditional Chinese Medicine, Zhejiang Hospital of Traditional Chinese Medicine, Zhejiang Chinese Medical University, Hangzhou 310006, China; 2 School of Life Sciences and Technology, Tongji University, Shanghai 200092, China; 3 Laboratory Diagnostics, Shanghai Changzheng Hospital, Shanghai 200003, China; 4 Department of Thoracic Surgery, First Hospital, College of Medicine, Zhejiang University, Hangzhou 310003, China

## Abstract

MicroRNAs are important gene regulators that potentially play a profound role in tumorigenesis. Increasing evidence indicates that miR-145 is a tumor suppressor capable of inhibiting breast and colon cancer cell growth both *in vitro *and *in vivo*. However, the biological function of miR-145 in non-small cell lung cancer (NSCLC) is largely unknown. In colon cancer cells, c-Myc is a confirmed direct target for miR-145. The aim of this work was to investigate the effect of miR-145 and c-Myc on proliferation of NSCLC cells, using the NSCLC cell lines A549 and H23 as models. We determined the expression level of miR-145 in tumor tissues relative to adjacent non-tumor tissues, and in NSCLC cell lines relative to non-malignant lung cells. Downregulation of miR-145 was seen in tumor tissues and the two NSCLC cell lines by real-time quantitative reverse transcription polymerase chain reaction. MTT and focus formation assays were conducted to measure cell proliferation rates. Cell growth was inhibited and the G1/S transition was blocked by miR-145 in transfection assays of A549 and H23 cells. We further showed that c-Myc was a direct target for miR-145. Introduction of miR-145 dramatically suppressed the c-Myc/eIF4E pathway, which was demonstrated to be crucial for cell proliferation in NSCLC cells. Furthermore, we found that CDK4 was regulated by miR-145 in cell cycle control. Taken together, our study results demonstrate that miR-145 inhibits proliferation of NSCLC cells through c-Myc. Increasing miR-145 expression may provide a novel approach for the treatment of NSCLC.

## Background

Lung cancer is the leading cause of cancer-associated deaths worldwide, and non-small cell lung cancer (NSCLC) accounts for almost 80% of lung cancer deaths [1,2]. Despite improvements in surveillance and clinical treatment strategies, the 5-year survival after curative resection is reported to be only 30-60% [3]. Thus, searching for rationally designed and targeted agents that mediate the initiation and progression of NSCLC and can be used for molecular targeted therapies is urgent and of great interest.

MicroRNA (miRNAs) are endogenously processed non-coding RNAs that regulate gene expression by blocking translation or decreasing mRNA stability [4,5]. Mature miRNAs comprise about 22 nucleotides, and are derived from longer pri-miRNA and pre-miRNA transcripts that undergo sequential processing by the RNase III-like enzymes Drosha and Dicer [6,7]. After maturation, miRNAs regulate gene expression by basepairing with mRNAs that are partially complementary to the miRNAs, generating miRNA-associated effector complexes. In contrast to small interfering (si)RNAs, miRNAs typically target a cluster of genes instead of one specific gene [8]. The binding of miRNAs to target mRNAs leads to translational repression or decreased mRNA stability. Emerging evidence shows that miRNAs have a variety of functions in regulation and in controlling cancer initiation and progression [9]. MiRNAs can function as tumor suppressors or oncogenes, depending on their specific target genes [10,11]. For example, miR-145, miR-335, miR-125b-1, miR-126, miR-15a, and miR-16-1 are all tumor suppressors for specific cancer types [12-15].

Recently, miR-145 was identified as a tumor-suppressive miRNA that is downregulated in several cancer types, including prostate cancer [16,17], bladder cancer [17], colon cancer [18-20] and ovarian cancer [21]. Accordingly, miR-145 overexpression has a growth inhibitory effect by targeting c-Myc [19] and IRS-1 [22]. In this study, we investigated the expression of miR-145 in NSCLC normal and tumor tissues, and in the NSCLC cell lines A549 and H23 and the non-malignant lung cell line Gekko Lung-1. We used overexpression of miR-145 to determine the effect on cellular proliferation and the cell cycle in A549 and H23 cells. We examined the effect of miR-145 on c-myc pathway protein expression and measured direct interaction by c-Myc binding. Moreover, c-myc, eIF4E and CDK knockdown inhibited cell proliferation of A549 and H23 cells. Furthermore, we demonstrated that CDK is crucial for cell cycle progression in A549 cells. Based on our results, we postulate that miR-145 inhibits NSCLC cell proliferation in part by mediating regulation of the c-myc/eIF4E pathway.

## Materials and methods

### Tissue collection

Paired NSCLC and adjacent non-tumor tissues were obtained with informed consent from 37 consecutive patients undergoing NSCLC resection surgery between July 2009 and March 2010 at Zhejiang Hospital of Traditional Chinese Medicine and Shanghai Changzheng Hospital, China. All tissue samples were flash-frozen in liquid nitrogen immediately after collection and stored at -80°C until use. Both tumor and non-tumor samples were confirmed by pathological examination. Patients were excluded if they had recurrent NSCLC or had primary NSCLC but received chemoradiotherapy before surgical operation [23].

### Cell culture

The human NSCLC cell lines A549 and H23 were from ATCC (ATCC# CCl-185, CRL-5800). Cells were cultured in Dulbecco's Modified Eagle Medium (DMEM; Sigma-Aldrich, St. Louis, Mo., USA) supplemented with 10% (vol/vol) fetal bovine serum (FBS) (Invitrogen, Carlsbad, CA, USA), 1% penicillin-streptomycin (v/v; 10,000 units/ml and 10,000 μg/ml, respectively; Invitrogen) and 1% Glutamax (v/v; Invitrogen). Cell cultures were incubated at 37°C in a humidified atmosphere containing 5% CO_2_. Stably transfected cells were cultured in the presence of 2 mg/ml puromycin (RocheH, Indianapolis, IN).

### Generation of stably transfected cell lines

Single-stranded DNA oligonucleotides with human *pre-miR-145 *(miRBase accession IDs MI0000461) sequences and with restriction enzyme site overhangs were from Integrated DNA TechnologiesH (Coralville, IA). Complementary sequences were annealed and the resulting double-stranded DNA was ligated to *Xho *I/*Not *I-digested pLemiR vector (Open Biosystems, Huntsville, AL). A549 cells were infected with plasmids using the Trans-Lentiviral GIPZ packing system (Open Biosystems; Huntsville, AL) according to the manufacturer's protocol. Briefly, TLA-HEK293TTM cells were transfected using Arrest-In with 37.5 μg plasmid DNA in serum-free medium for 4 h. Media was then replaced with serum-containing media for 36 h. Media were collected, centrifuged to remove cell debris and used to infect A549 and H23 cells. At 48 h after addition of virus, infected cells were selected by adding 2 mg/ml puromycin.

### Real-time RT-PCR (qPCR) for small RNA quantification

Total RNA (20 ng), isolated using a PureLink Micro-to-Midi total RNA isolation kit (Invitrogen) according to the manufacturer's protocol, was reverse transcribed using a TaqMan reverse transcription (RT) kit (Applied Biosystems, Foster City, CA) and RNA-specific primers with TaqMan microRNA assays (Applied Biosystems) in 15 μl, with annealing at 16°C for 30 min followed by extension at 42°C for 30 min. From the RT reaction, 1.33 μL was combined with 1 μL specific primers for either *RNU6B *or *miR-145 *(Applied Biosystems, Foster City, CA) in triplicate wells for 44-cycle PCR using a 7900HT thermocycler (Applied Biosystems). Denaturation at 95°C was for 15 s, and annealing and extension at 60°C was for 1 min. SDS software (Applied Biosystems, Foster City, CA) was used to determine cycle-threshold (Ct) fluorescence values. Prism 5.0b software (GraphPad; La Jolla, CA) was used for statistical analysis and graphing.

### c-Myc luciferase reporter assay

Cultures were transfected with 5 μg, 10 μg, or 15 μg pBV-c-Myc-luc plasmid using Metafectene Pro. The next day, cells were replated and incubated overnight. Cultures were treated as indicated for 24 h and luciferase activity was determined using a luciferase kit (Promega), normalizing to protein concentration and then to a control sample transfected with pBV-luc and treated with DMSO.

### Cell viability analysis and focus formation assay

Cell proliferation was evaluated by 3-(4,5-dimethylthiazol-2-yl)-2,5-diphenyl tetrazolium bromide (MTT) assay. Briefly, cells were plated in 96-well plates with 4000 cells in 100 μl per well and incubated for 72 h. MTT was added under sterile conditions, and the cells were incubated for 4 h before reading absorbance at 570 nm in an enzyme-linked immunosorbent assay plate reader. Each experiment was performed in six replicate wells and independently repeated three times. Absorbance values were normalized to media control. For focus formation assays, cells transfected with vector, or cells expressing miR-145 were seeded on 35-mm dishes at 60-80% confluence. After 24 h, cells were trypsinized and split into six-well dishes as described previously [24].

### Transient expression of CDK4

Cells were transfected with 5 μg human wild-type (Wt) pCMV-cdk4 using Metafectene Pro transfection reagent (Biontex) according to the manufacturer's protocol. After 24 h, cells were replated and cultured for 24 h before measurement.

### Cell cycle analysis

Cells grown to 70%-90% confluence were detached by trypsinization, fixed in 70% ethanol at 4°C for 1-2 days, washed with phosphate-buffered saline (PBS), and incubated at a density of 1-2 × 10^6 ^cells/ml with 0.3 μM 4,6-diamidino-2-phenylindole dihydrochloride (DAPI; MP Biochemicals, Solon, OH) in PBS at room temperature in the dark for 100 min. After washing once with PBS, DAPI fluorescence was assayed using an LSR II (BD Biosciences, San Jose, CA) flow cytometer equipped with a 408-nm violet laser diode and a 450/50 nm emission filter.

### Western blot analysis

To determine protein expression levels, cells were harvested and lysed in RIPA lysis buffer (50 mM Tris-HCl, pH 8.0, 150 mM NaCl, 0.1% SDS, 1% NP-40, 0.25% sodium deoxycholate and 1 mM EDTA) with freshly added protease inhibitor cocktail (Roche) for 15 min on ice, then centrifuged at 13,000 rpm for 10 min. Total protein of clarified supernatants was quantified by bicinchoninic acid assay (BCA) kit (Pierce Biotechnology). To analyze protein levels, blots were blocked with 5% milk in PBST (0.05% Tween-20 in PBS) and probed with primary antibody against c-Myc (1:500), eIF4E (1:1000), CDK4 (1:2000), or beta-actin (1:1000) for 4 h at room temperature or overnight at 4°C, and followed by horseradish peroxidase-conjugated secondary antibody (Pierce, Rockford, IL) for 1 h at room temperature. Protein bands were detected with SuperSignal West Pico chemiluminescence substrate (Pierce) and processed with the GenTools software package. In each experiment, the same amount of protein was used, and the experiments were repeated independently at least three times.

### Chromatin immunoprecipitation (ChIP) assays

ChIP assays were performed using a ChIP Assay Kit (Upstate Biotechnology, Lake Placid, NY, USA) on A549 cells cultured to 70-80% confluence. Chromatin was cross-linked with 1% formaldehyde at 37°C for 10 min. Cells were washed with cold PBS twice and disrupted in SDS lysis buffer containing protein inhibitor cocktail. Chromatin was sonicated to an average length of 200 to 1000 bp as verified by agarose gel. Sonicated cell supernatants were diluted 10-fold in ChIP dilution buffer containing protein inhibitor cocktail and an aliquot was reserved for input control. Antibody against c-Myc (10 μg, Abcam, Cambridge, MA) was added and the chromatin solution was gently rotated overnight on ice. Protein A agarose slurry was added and incubated at 4°C for 1 h with constant rotation. Agarose beads were collected by centrifugation and washed, and antibody-bound chromatin released from the agarose beads. DNA was purified by phenol/chloroform extraction and ethanol precipitation. Binding was detected by PCR. A 10-kb region downstream from the binding site was used as a negative control.

### shRNA transfection

ShRNA constructs against c-Myc, eIF4E and CDK4 were from Origene Company (Rockville, MD). A549 or H23 cells were cultured until 70%-80% confluence. Cells were transfected with shRNA using transfection reagent Fugene HD (Roche) according to the manufacturer's instructions. The level of miR-145 expression was determined using PCR.

### Statistical analysis

All data are presented as mean ± standard deviation (SD). Statistical significance was determined by two-tailed Student's *t*-test. *P*-values of < 0.05 were considered statistically significant. Analyses used GraphPad Prism version 5.0 for Windows, GraphPad Software (San Diego, CA).

## Results

### Expression profile of miR-145 in non-small cell lung cancers

**Figure 1:**
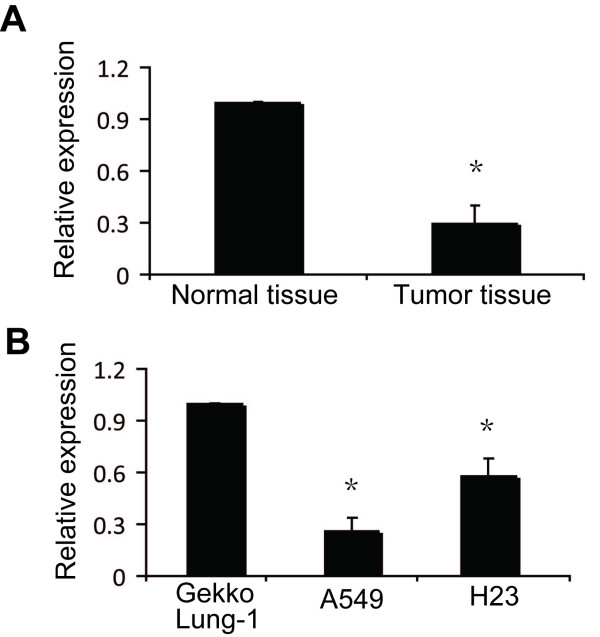
**Expression of miR-145 in normal tissues and non-small cell lung cancer**. miR-145 levels were measured by miRNA TaqMan qRT-PCR in normal and in NSCLC tissue (A), and in the normal lung cell line Gekko Lung-1, and the NSCLC cell lines A549 and H23 (B). (A) Relative levels of miR-145 were lower in tumor tissue than in normal tissue. (B) Relative levels of miR-145 in the NSCLC cell lines, particularly A549, were lower than in Gekko Lung-1 cells. Vertical axis indicates relative expression of each miRNA normalized to control. Results are mean ± SD of three independent experiments. *P < 0.05 by Student's paired *t*-test compared to untreated cells (control).

Prompted by numerous reports of miR-145 downregulation in cancer [25-27], we sought to identify the role of miR-145 in NSCLC. We compared the expression levels of miR-145 in NSCLC compared to corresponding normal tissues by qPCR for miR-145 in 37 matched pairs of tumor and non-tumor tissues from patients. We also measured expression in a non-tumorigenic lung cell line and two human NSCLC cell lines. As shown in Figure 1A, miR-145 expression levels were significantly decreased in tumors compared to the paired normal samples. Further, compared to the normal lung cell line Gekko Lung-1, the NSCLC cell line A549 showed about 80% significantly lower expression of miR-145, and H23 NSCLC cells showed approximately 50% lower expression (Figure 1B). The reduced expression of miR-145 in NSCLC suggested that miR-145 is a potential anti-oncogenic miRNA in this cancer.

### miR-145 overexpression inhibits the proliferation of human NSCLC cells

**Figure 2:**
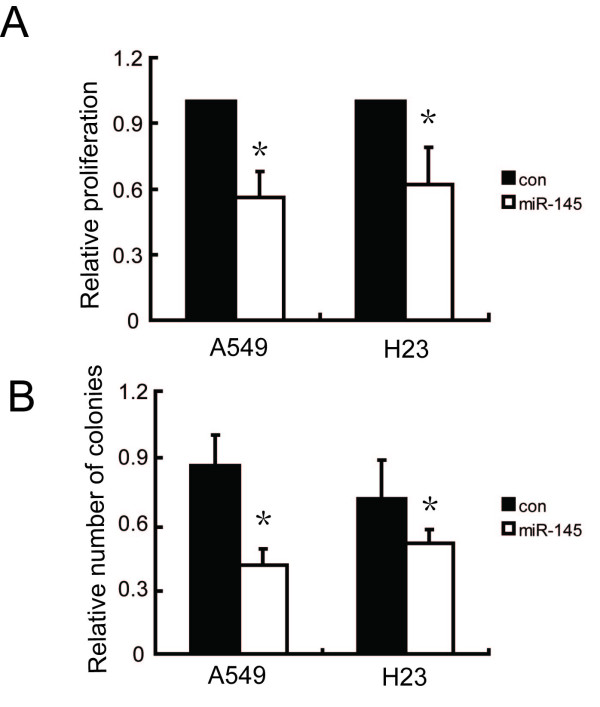
**miR-145 overexpression reduces the proliferative potential of A549 and H23 cells**. (A) MTT assays reveal reduced cell growth for stable transfected cell lines compared to vector-transfected control. (B) Methylene blue-stained culture plates demonstrated no difference in adherent colony formation in six-well dishes. Values are means of three separate experiments ± SD. *P < 0.05 by Student's paired *t*-test compared to untreated cells (control).

To test the function of miR-145 in cell growth, we used miR-145 precursor miRNA to infect human NSCLC A549 and H23 cells, both of which showed good transfection efficiency. After transfection, miR-145 levels were increased in both cell lines, indicating that enhancement was due to the introduction of precursor miR-145 (data not shown). As demonstrated by MTT growth assays, overexpression of miR-145 dramatically reduced cell proliferation in both cell lines (Figure 2A). To assess biological activity, focus formation assays were performed on A549 and H23 cells. Compared to cells transfected with control vector, the number of colonies from A549 and H23 cells overexpressing miR-145 decreased by about 50% and 15%, respectively (Figure 2B).

### miR-145 regulates cell-cycle progression

**Figure 3:**
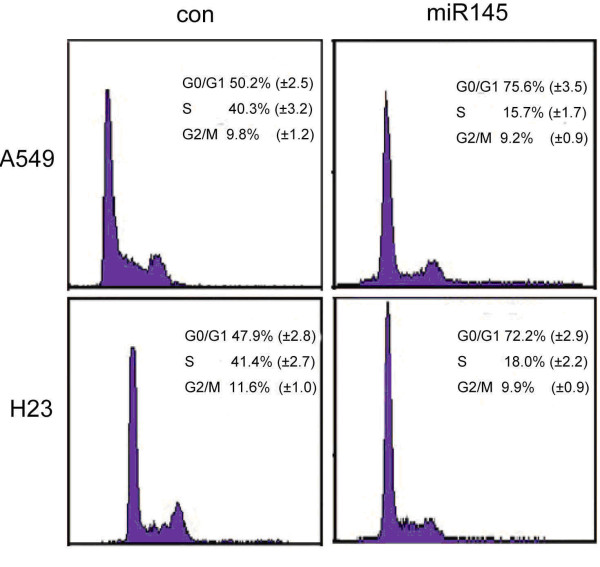
**Effect of miR-145 on A549 and H23 cell cycle**. A549 and H23 cells were stablely transfected with vector control or *miR*-*145 *expression vector. After 2 days, cells were harvested for cell cycle analysis. (A) Percentage of A549 cells transfected with vector control or miR-145 expression vector at different phases. (B) Percentage of H23 cells transfected with vector control or miR-145 expression vector cells at different phases. Data were obtained by densitometry measurement and the mean of three experiments.

Cell cycle analysis results showed a significant decrease in growth after transfection to overexpress miR-145, indicating that cell proliferation was inhibited. In addition, we found that cells transfected to overexpress miR-145 accumulated in G1 phase. This suggested that miR-145 regulates cell-cycle progression primarily by delaying the G1/S transition (Figure 3).

### miR-145 directly targets the c-Myc/eIF4E pathway in human NSCLC A549 cells

**Figure 4:**
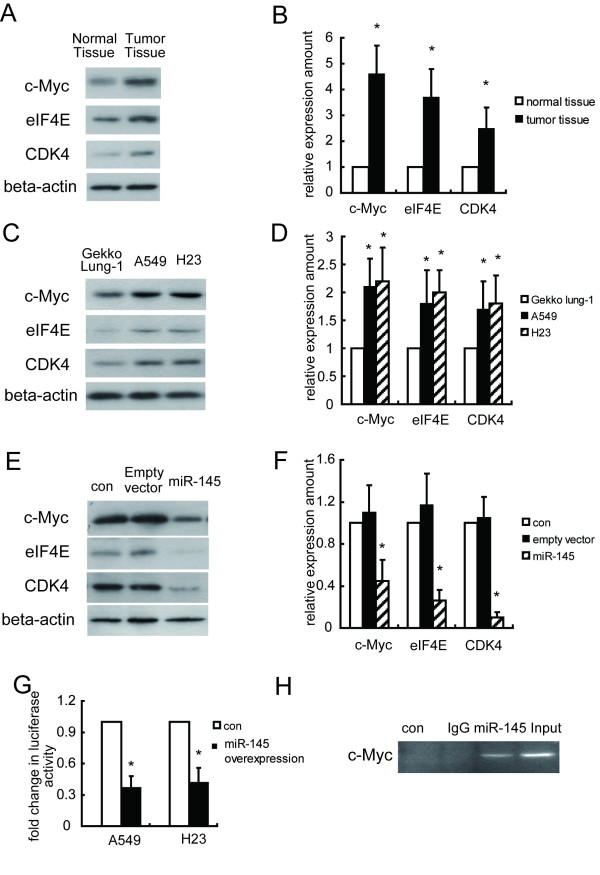
**miR-145 regulates the c-myc/eIF4E pathway in NSCLCs**. Western blot analysis of c-myc, eIF4E, and CDK4 expression levels in normal and tumor tissue (A, B), and one normal lung cell line and two NSCLC cell lines (C, D). (E, F) Western blot for c-myc, eIF4E, and CDK4 after transfection with pre-miR-145 expression vector and or control miRNA vector. (G) Cells transiently transfected with the empty pBV-luc plasmid vector or pBV-c-Myc-luc plasmid were treated for 24 h. Luciferase activity was normalized to protein concentration and then to measurements from pBV-luc-transfected, DMSO-treated control cultures. (H) ChIP assays of c-Myc binding to miR-145 DNA. The beta actin gene was used as an internal control.

c-Myc induction of eIF4E in cancer cells is frequently used as a cellular model to study the molecular mechanism of cancer proliferation, but its interaction with the expression signature of miR-145 is not completely understood. Therefore, we investigated if miR-145 directly regulated the c-Myc/eIF4E pathway. Examination of 37 paired tissues of NSCLC tumors and adjacent uninvolved lung, and the NSCLC cell lines for c-Myc, eIF4E and CDK4 expression showed enhanced levels in tumor tissues and cancer cell lines (Figure 4A-D). We confirmed that miR-145 downregulated c-Myc and the c-Myc target genes eIF4E and CDK4, which are involved in cell proliferation and cycle regulation (Figure 4E, F). We further investigated if miR-145 directly regulated the c-Myc/eIF4E pathway by luciferase assay and found that overexpression of miR-145 reduced c-Myc levels. (Figure 4G). ChIP analysis using specific c-Myc antibody and PCR of the precipitated DNA with a primer set confirmed the physical association of c-Myc with the endogenous miR-145 promoter in A549 cells (Figure 4H). In contrast, a non-specific primer set to amplify a region 11 kb downstream of the miR-145 promoter did not produce a PCR product.

### Suppression of c-Myc, eIF4E and CDK4 inhibit proliferation of A549 and H23 cells

**Figure 5:**
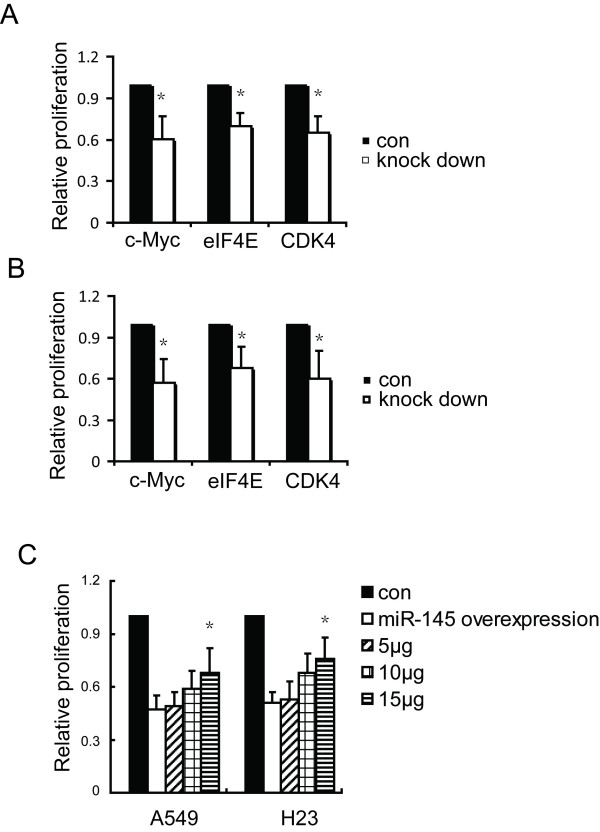
**Suppression of c-myc, eIF4E, andCDK4 by RNAi reduces A549 and H23 proliferation**. (A) Suppression of cell proliferation by c-myc, eIF4E and CDK siRNA in A549. (B) Suppression of cell proliferation in H23, as in part (A). A549 and H23 cells were transfected with c-myc, eIF4E and CDK siRNA, and assayed by MTT. (C) A549 and H23 cells were transiently transfected with vector control, *miR*-*145 *expression vector or miR-145 expression vector plus pCMV-CDK4, followed by MTT assay. Data are mean ± SD of three independent experiments. *P < 0.05 by Student's paired *t*-test compared to untreated cells (control).

Previous studies have shown that c-Myc/eIF4E is important in cellular proliferation and protein synthesis [28]. Thus, increased levels of c-Myc/eIF4E might function in the growth advantage of tumors. To investigate the biological significance of c-Myc, eIF4E, and CDK4 in NSCLC cells, we tested whether RNAi-mediated reduction of c-Myc, eIF4E and CDK4 levels influenced the growth rate of A549 and H23 cells. We found that silencing expression of c-Myc, eIF4E, or CDK4 significantly decreased the growth rate of A549 and H23 cells by 35%-45% in three separate experiments (Figure 5). Overexpression of CDK4 by transfection of a Wt pCMV-CDK4 vector into NSCLC cell lines rescued the growth inhibition induced by elevated expression of miR-145.

### miR-145 regulated CDK4 is crucial for cell cycle progression in A549 cells

**Figure 6:**
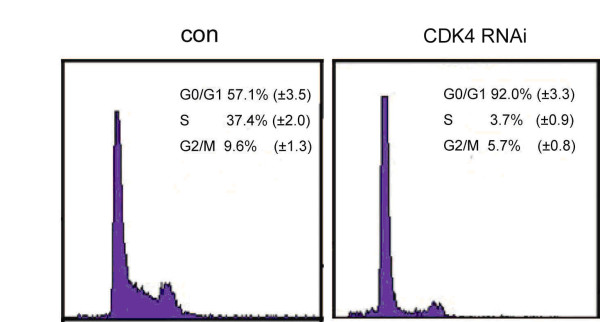
**CDK knockdown by RNAi induces cell cycle arrest in A549**. Percentage of A549 cells transfected with vector control or CDK siRNA at different phases, by cell cycle densitometry measurement. Data are the mean of three experiments.

Cell cycle analysis determined that the effect of miR-145 on cell proliferation of NSCLC cells was due to cell cycle alterations. We tested whether RNAi-mediated reduction in eIF4E or CDK4 levels influence the cell progression of A549 cells and found that RNAi directed against CDK4 resulted in an increase in the percentage of cells in G1 phase from 60.7% to 92.5% (*P *< 0.01) (Figure 6). However, knockdown of eIF4E by siRNA did not alter cell cycle progression of A549 cells. These results indicated that downregulation of CDK4 by miR-145 induced a G1 cell-cycle arrest in NSCLC cells.

## Discussion

MiRNAs are frequently deregulated in malignant tissues [29]. Recently, the expression of miRNAs such as let-7 and miR-126 were found to be frequently reduced in lung cancer, both *in vivo *and *in vitro*, and reduced expression was significantly associated with shortened postoperative survival, independent of disease stage [30-32]. We studied the expression profile of miR-145, which is underexpressed in several tumor types [18,33] and found that *miR*-*145 *was underexpressed in NSCLC specimens compared to matched normal tissue samples (Figure 1A), and was drastically reduced in NSCLC cell lines compared to the non-malignant lung cell line Gekko Lung-1. This suggested miR-145 is a potential tumor suppressor in NSCLC. Downregulation of miR-145 was more prominent in A549 cells than in H23 cells, indicating variability of this effect in different cell lines. These findings prompted us to investigate the regulation of *miR*-*145 *in NSCLC cells, since differential expression of miRNAs suggests that miRNAs may be involved in the genesis and development of tumors.

To characterize the biological effects of miR-145 in tumor cells, we employed the NSCLC cell lines A549 and H23. In agreement with reports showing a growth inhibitory effect of miR-145 [19,34], we also observed a significant growth reduction of A549 and H23 cells upon transfection with an miR-145 expression vector, and the most pronounced growth inhibitory effect was seen in A549 cells. We investigated the effect of miR-145 in the progression of cell cycle and showed that lentivirus-mediated expression of miR-145 induced cell cycle arrest. MiR-145 overexpressing cells showed G0/G1 phase arrest and S-phase reduction in both cell lines, suggesting that the reduced growth of the cells may be associated with cell cycle arrest.

Recent studies showed that miR-145 silenced c-Myc and its downstream targets in colon cancer, which be associated with c-Myc/eIF4E as a miR-145 target [19]. Interestingly, downregulation of the miR-145 in NSCLC is consistent with upregulation of c-Myc, eIF4E and CDK4 in the same sample set which is consistent with our finding that c-Myc is a major target for miR-145 by ChIP. Knock down of c-Myc, eIF4E and CDK4 respectively showed that they are all important for proliferation in both cell lines. Furthermore, by silencing eIF4 and CDK4 we confimed CDK4 is crucial in the progression of cell cycle. Based on our findings, we propose that miR-145 regulates NSCLC cell proliferation partly by targeting c-Myc, and that the loss of miR-145 may provide a selective growth advantage during lung carcinogenesis.

In summary, we conducted miR-145 expression profiling in human NSCLC cells, and focused on the identification of targets of abnormally expressed miR-145. Our results showed that miR-145 was significantly downregulated and might be used as a marker for advanced NSCLC. In addition, we also found that miR-145 targeted c-Myc, which suggested an explanation for the carcinogenesis pathway mediated by miR-145 and provided data that may contribute to molecular targeted therapy based on miRNAs.

## Competing interests

The authors declare that they have no competing interests.

## Authors' contributions

ZC carried out cell cyle determination and preparing the draft. HZ carried out the immunoassays. YG participated in the immunoassays. YG did the cell proliferation assay. AD and JH participated in the design of the study and performed the statistical analysis. LP and WAN conceived of the study, and participated in its design and coordination. All authors read and approved the final manuscript.
